# Determining hypertensive patients’ beliefs towards medication and associations with medication adherence using machine learning methods

**DOI:** 10.7717/peerj.8286

**Published:** 2020-03-13

**Authors:** Firdaus Aziz, Sorayya Malek, Adliah Mhd Ali, Mee Sieng Wong, Mogeeb Mosleh, Pozi Milow

**Affiliations:** 1 Bioinformatics Science Programme, Institute of Biological Sciences, University of Malaya, Kuala Lumpur, Malaysia; 2 Quality Use of Medicines Research Group, Faculty of Pharmacy, Universiti Kebangsaan Malaysia, Kuala Lumpur, Malaysia; 3 Software Engineering Department, Faculty of Engineering & Information Technology, Taiz University, Taiz, Yemen; 4 Environmental Management Programme, Institute of Biological Sciences, University of Malaya, Kuala Lumpur, Malaysia

**Keywords:** Random forest, Artificial neural network, Self-organizing Map (SOM), Hypertension, Support Vector Regression, Variable importance, Adherence level

## Abstract

**Background:**

This study assesses the feasibility of using machine learning methods such as Random Forests (RF), Artificial Neural Networks (ANN), Support Vector Regression (SVR) and Self-Organizing Feature Maps (SOM) to identify and determine factors associated with hypertensive patients’ adherence levels. Hypertension is the medical term for systolic and diastolic blood pressure higher than 140/90 mmHg. A conventional medication adherence scale was used to identify patients’ adherence to their prescribed medication. Using machine learning applications to predict precise numeric adherence scores in hypertensive patients has not yet been reported in the literature.

**Methods:**

Data from 160 hypertensive patients from a tertiary hospital in Kuala Lumpur, Malaysia, were used in this study. Variables were ranked based on their significance to adherence levels using the RF variable importance method. The backward elimination method was then performed using RF to obtain the variables significantly associated with the patients’ adherence levels. RF, SVR and ANN models were developed to predict adherence using the identified significant variables. Visualizations of the relationships between hypertensive patients’ adherence levels and variables were generated using SOM.

**Result:**

Machine learning models constructed using the selected variables reported RMSE values of 1.42 for ANN, 1.53 for RF, and 1.55 for SVR. The accuracy of the dichotomised scores, calculated based on a percentage of correctly identified adherence values, was used as an additional model performance measure, resulting in accuracies of 65% (ANN), 78% (RF) and 79% (SVR), respectively. The Wilcoxon signed ranked test reported that there was no significant difference between the predictions of the machine learning models and the actual scores. The significant variables identified from the RF variable importance method were educational level, marital status, General Overuse, monthly income, and Specific Concern.

**Conclusion:**

This study suggests an effective alternative to conventional methods in identifying the key variables to understand hypertensive patients’ adherence levels. This can be used as a tool to educate patients on the importance of medication in managing hypertension.

## Introduction

Hypertension is one of the most significant cardiovascular risk factors and the leading cause of mortality worldwide. Malaysia is facing an upsurge in the prevalence of hypertension among adults ages 18 and older, reportedly 32.7% (Institute for Public Health, Ministry of Health, Malaysia, 2011). The main reason identified for uncontrolled hypertension levels is non-adherence to prescribed treatments (Abdul-Razak et al., 2016; Ross, Walker & MacLeod, 2004; Wang et al., 2002). If left untreated, hypertension can lead to severe complications such as cardiovascular, cerebrovascular, and kidney diseases (Khatib & EI-Guindy, 2005), making adherence to hypertensive medication vital when controlling this condition. Understanding the factors associated with medication adherence is essential in reducing unnecessary costs from changes in treatment regimens and from further medical complications, which may lead to hospital admission and prolonged stays.

Ross, Walker & MacLeod (2004) reported that patients’ beliefs about diseases and therapies affected their adherence to treatment. These beliefs are usually influenced by a combination of several factors, such as demographics, personalities, and knowledge of the disease and therapy regimens (Glanz, Rimer & Viswanath, 2008). Additionally, it was found that patients’ understanding regarding their health condition and therapy could lead to changes in their beliefs about their disease and its regimen therapy (Magadza, Radloff & Srinivas, 2009).

Currently, the Belief about Medicines Questionnaire (BMQ) is used to measure perceptions toward medications among patients suffering from common chronic diseases such as hypertension (Ruppar, Dobbels & Geest, 2012). The BMQ divides medication beliefs into two sections: General beliefs (Overuse and Harm) and Specific beliefs (Necessity and Concerns). The BMQ questionnaire assesses medication beliefs in general and in specific situations, and permits the investigation of general perceptions of medication in both situations (Horne, Weinman & Hankins, 1999). Statistical analysis techniques are commonly used for the evaluation of adherence towards hypertension medication (Venkatachalam et al., 2015; Krousel-Wood et al., 2010). However, because of the large amount of data continuously acquired in the medical and pharmaceutical fields, predictive models developed using machine learning methods are being used to extract knowledge and identify relationships from the data. Some machine learning methods are also known as ensemble learning methods. The learning process involves multiple learners using the data to solve a problem related to the acquired data (Li & Zhou, 2007). Standard machine learning algorithms used in hypertension-related studies are Random Forests (RF), Artificial Neural Networks (ANN), Support Vector Machines (SVM) and Self-Organizing Feature Maps (SOM). However, Support Vector Regression (SVR) was implemented in this study because it has the same principle as SVM for classification cases. SVR is suitable when the response variable is numerical rather than categorical (Vapnik, Golowich & Smola, 1997).

Farran et al. (2013) successfully applied machine learning and statistical methods, namely SVM, logistic regression and k-nearest neighbours (k-NN), to determine the risk of hypertension. Hypertensive-related studies typically use machine learning without comparison with conventional statistical methods. Lee et al. (2013) used SVM to determine medication adherence for chronic diseases. Son et al. (2010) applied SVM to predict medication adherence in heart failure patients. Among hypertensive patients, ANN has been used to diagnose hypertension and to predict salt intake reduction behaviour (Kaur & Bhardwaj, 2014). RF has been used in predicting the risk of chronic diseases (e.g., hypertension) from a medical diagnosis history (Khalilia, Chakraborty & Popescu, 2011) and diabetic retinopathy classification analyses (Casanova et al., 2014). SOM, an unsupervised type of machine learning method, allows the detection of relationships in and the visualisation of higher-dimensional non-linear data. Kihato (2013) used SOM in the analysis and visualisation of metabolic syndrome.

However, few publications have reported on the application of machine learning methods in determining parameters associated with adherence among hypertensive patients. Determining adherence levels is an essential complex matter that should not be measured as a dichotomous parameter (adherent versus non-adherent) (Vrijens et al., 2017). Adherence level representing the precise numerical score is vital in differentiating between patients who scored poorly and the others, and in creating an appropriate intervention program tailored to the specific needs of a group of patients. Hence, this study aimed to assess the feasibility of regression types of machine learning methods in determining adherence levels that produce an exact value for adherence outcomes. RF, ANN and SVR were used to predict adherence levels, and SOM was used for the visualisation of variables associated with hypertensive patients. The objective of this study was to compare the performances of machine learning methods in predicting hypertension medication adherence. The study also aimed to determine and visualize the relationship between significant variables associated with hypertension medication adherence.

## Materials & Methods

### Ethics approval

The Clinical Research Ethics of UKMMC approved this study with the registration number UKM 1.5.3.5/244/SPP/NF-023-2011.

### Data collection and preparation

A total of 160 patients enrolled in this study. Patients were recruited through the outpatient clinics at the University Kebangsaan Hospital (UKM) in 2011.

A principal investigator approached and invited patients to participate in this study. All adult patients diagnosed with essential hypertension, and hypertensive patients who had been on at least one antihypertensive medication for more than one year were considered eligible. Exclusion criteria for patient selection included gestational hypertension, a diagnosis of another concomitant terminal disease, and difficulty communicating. Those who agreed to participate in this study signed the consent forms before enrolment and filled out the questionnaire with the investigator present. The questionnaire was collected and checked upon completion.

The questionnaire was developed based on the validated questionnaires BMQ and a conventional medication adherence scale, Malaysian Medication Adherence Scale (MALMAS), which enables an analysis of overall perceptions of medication. The BMQ is an 18-item questionnaire which measures medication beliefs in general (BMQ-General) and medication beliefs for specific situations such as chronic illnesses (BMQ-Specific). The BMQ-General covers eight items composed of two scales: (a) General Harm measures beliefs about how harmful medicines are (harm scale) and (b) General Overuse addresses the concept of over-prescription by doctors who place too much confidence in medication (belief scale). Each scale consists of four items, with total scores ranging from 4 to 20. Higher scores indicated more negative beliefs towards medicines in general (Menckerberg et al., 2008). The BMQ-Specific was comprised of a Specific Necessity scale and a Specific Concerns scale. The Specific Concerns scale evaluates the possibility of adverse reactions resulting from consuming the prescribed medication. The Specific Necessity scale looks into the patient’s belief about their individual requirements in adhering to their prescribed medicine. Each scale consists of five items. Scores obtained for the individual items were summed up with total scores ranging from 5 to 25. Higher scores in the General Harm and General Overuse categories indicate a negative perception of the medication.

Similarly, higher scores obtained in the Specific Concerns category signify that adverse reactions are believed to be possibly harmful with regular intake of medication. Higher scores in the Specific Necessity category indicate the patient’s need to adhere to medication to preserve good health (Horne, Weinman & Hankins, 1999; Menckerberg et al., 2008). A necessity-concerns differential (NCD) was calculated by subtracting the concerns scores from the necessity scores. This score was used to assess the balance between perceived benefits (Specific Necessity) and costs (Specific Concerns) regarding the prescribed medication. Positive differences indicated that the necessity of medication outweighed the concerns, and negative differences indicated the opposite (Horne & Weinman, 1999).

Validated patients self-reported their medication adherence using the medication adherence scale. This scale has total scores ranged from 0 to 8. Adherence was considered high if patients had an overall score anywhere between 6 and 8, and adherence was considered low if they had a total score from 0 to 5 (Chua et al., 2013).

Table 1 presents summary statistics for the categorical and continuous variables used in this study. The measured variables were obtained from the questionnaire and divided into three categories: demographic characteristics of the patient, history of medication and disease, and beliefs towards medications.

**Table 1:** The summary statistics of all the variables.

**Variable**	**Attributes**	**Value**	**Percentage (%)**
Age	Mean ± SD	65 ± 9	–
	Age range	42-87	–
	Median	65	–
Gender	Male	113	70.6
	Female	47	29.4
Ethnicity	Malay	56	35.0
	Chinese	93	58.0
	Indian	11	7.0
Religion	Islam	60	37.5
	Buddha	63	39.4
	Hindu	10	6.3
	Christian	17	10.6
	Others	10	6.3
Educational level	Primary	50	31.3
	Secondary	71	44.4
	Tertiary	21	13.1
	Degree	10	6.3
	Masters	5	3.1
	Doctor of philosophy	3	1.8
Occupational field	Agricultural	0	0.0
	Business	6	3.8
	Education	3	1.9
	Health	1	0.6
	Housework	5	3.1
	Engineering	2	1.3
	Unemployed	24	15.0
	Retiree	93	58.1
	Others	26	35.0
Monthly income	<RM1000	108	67.5
	RM1000–RM2000	23	14.4
	RM2001–RM3000	6	3.8
	RM3001–RM4000	10	6.3
	RM4001–RM5000	6	3.8
	>RM5000	7	4.4
Marital status	Single/Widow/Widower	17	10.6
	Married	143	89.4
Duration of antihypertensive medications intake	1–4 years	46	28.8
	5–10 years	36	22.5
	>10 year	78	48.8
Presence of other concomitant diseases	Yes	122	76.2
	No	38	23.8
Total number of antihypertensive medications taken per day	Range medicine	0.5–23	–
Aids in antihypertensive medications intake	Pillbox	109	68.0
	Timetables	10	6.3
	Others	41	25.6
Counseling for antihypertensive medications	Yes	100	62.5
	No	60	37.5
Specific necessity	Mean ± SD	17.3 ± 2.8	–
Specific concern	Mean ± SD	13.0 ± 4.8	–
General overuse	Mean ± SD	10.8 ± 1.8	–
General harm	Mean ± SD	7.6 ± 2.2	–
Adherence level	Mean ± SD	4.3 ± 1.7	–

### Model development

In this study, three different ML methods, RF, ANN and SVR, were implemented and compared to determine hypertensive medication adherence levels. Prior to model development, variable significance using RF feature importance was determined. The RF feature importance ranked input variables based on their importance in medication adherence. Feature selection was performed based on the ranked variables using the backward elimination method on a trained RF model to identify significant variables associated with medication adherence. The RF, SVR and ANN models were then constructed using the features selected from the feature selection method and all input variables for comparison. Model validation and performance evaluation were carried out to avoid overfitting and biases in the results. SOM was also used in this study to visualize and understand the relationships between input variables and medication adherence.

### Model tuning, training and testing

Data normalisation was performed before model development as some variables have a more substantial variation or spread. The normalisation of the raw datasets, therefore, was necessary to ensure that all variable values were within the same range. Normalization is essential for machine learning models such as ANN and SVR (Ogasawara et al., 2010; Shen et al., 2016). Ten-fold cross-validation was used as a resampling procedure to evaluate machine learning models on a limited data sample (Geisser, 1993), which was implemented using the R caret package. Application of *K*-fold cross-validation results in a less biased or less optimistic estimate of the model performance compared to simple methods such as train or test split (Kim, 2009). The output was then de-normalized before evaluating the model performance.

Machine learning model performance assessment was performed using the root mean square error (RMSE). The RMSE was calculated based on the de-normalized value of the model output. It was also used to measure the average level of prediction error, and indicates the ideal fit of the model to the data and how close the observed data points are to the model’s predicted values (Armstrong & Collopy, 1992).

The Wilcoxon Signed-Rank test is a nonparametric test that can be used to decide if ranks differ between matched samples (Lowry, 2013). The Wilcoxon Signed-Rank test is preferred to the *t*-test, which is suitable only when there is a normal distribution of differences (Shier, 2004). The Wilcoxon Signed-Rank test omits signs and compares the ranks for positive and negative differences. Differences are ranked based on their absolute values (in case of a tie, average ranks are computed), and the positive and negative sum of the ranks are calculated (Wilcoxon, 1945).

### Algorithms

A Random Forest is an ensemble method comprised of a random number of trees used to determine the outcome (Breiman, 2001; Liaw & Wiener, 2002). A subset is randomly chosen from the full set of predictors, *p*, at each tree node, which is denoted by mtry (Díaz-Uriarte & De Andres, 2006). RF uses the Gini index node of impurity calculated based on a set of predictors to select the best split at each node (Khalilia, Chakraborty & Popescu, 2011). Test set error is determined from the Out of Bag (OOB) error generated from a tree grown from a bootstrap dataset, and is subsequently used to estimate variable importance. Variable importance is a useful by-product of the RF algorithm (Verikas, Gelzinis & Bacauskiene, 2011). In RF for regression, the test error estimate is defined by the RMSE. The RF algorithm implemented in this study was based on Breiman (2001). Varying the value of mtry and the number of trees (ntree) in this study determined the optimum RF model with the best results. An mtry value of 4 and ntree value of 3000 provided the best results with the lowest error rates for the RF prediction model.

The Resilient Backpropagation (Rprop) algorithm (Riedmiller & Braun, 1992) was used in this study for the ANN model development. This algorithm uses the positive or negative sign of the gradient to illustrate the direction of the adjustment weight. The architecture of the ANN used in this study was determined through trial and error. A logistic transformation function was used with one hidden layer, and the learning rate value was set to 0.01. The ANN network geometry constructed for all and selected variables consisted of five neurons in the hidden layer.

SVR applied in this study had the same principle as SVM for classification cases; SVR is suitable when the response variable is numerical rather than categorical (Vapnik, Golowich & Smola, 1997). SVR is a non-parametric technique that depends on kernel functions and uses the principle of maximal margin as a convex optimization problem. SVR uses a cost parameter to avoid over-fit. The cost parameter was set to the value of *C* = 1 in this study. In this study, the SVR model was built using the Radial Basis Function (RBF) kernel.

SOM (Michalski, Carbonell & Mitchell, 1983) was used in this study to ordinate factors associated with adherence level. The Euclidian distance between the input factors was calculated and visualized as a *U*-matrix (unified distance matrix) and component plane as a result of the training of this unsupervised ANN. SOM reduces data dimensions and displays similarity by producing one or two dimensions and grouping similar inputs together. A component plane illustrates the comparative values of one component of the codebook vectors, and the u-matrix visualizes the distances between the codebook vectors in a two-dimensional map (Kohonen, 2001). The SOM is coloured by the values of U-matrix elements. A dark colour (red) between the neurons represents a large distance. A light colour (blue) between the neurons signifies that the codebook vectors are close to each other in the input space. Light areas signify clusters and dark areas are cluster separators (Stefanovic & Kurasova, 2011). The quality of the SOM map was evaluated using topological and quantisation errors.

### Feature selection

Feature selection is the process of ranking variables and identifying and eliminating irrelevant and redundant information. This process of dimensionality reduction enables machine learning algorithms to operate faster. The RF variable importance method was used for feature selection in this study. This method was essential in determining significant variables associated with hypertensive patients’ adherence levels. The variables were ranked in descending order based on the OOB error rate, starting with the most important variable with the largest increase in the mean percentage of the error, and ending with the least important variable with the smallest increase in the mean percentage of error. Backward elimination was carried out based on the ranked variables in ascending order. The error rate was calculated after each elimination step. If the error rate increased, the eliminated variable was considered significant or essential to medication adherence.

Variables selected from this process were introduced to all other ML algorithms used in this study. The performances of these models were then compared against RF, SVR and ANN models developed using all input variables.

### Additional statistics

The results were expressed as mean and SD for continuous variables, and as frequencies for categorical variables. Correlation analysis was carried out to identify significant relationships between variables.

### Software

R software (Version 3.5.2) was used in this study for the development of RF, ANN and SVR models. The following functions were used: randomForest (Version 3.5.2), neuralnet (Version 3.5.2), and caret (Version 3.5.2). SOM was developed using MATLAB (version 16b). Statistical Package for Social Sciences (SPSS) program version 16.0 was used for statistical analysis and data cleaning.

## Results

The variables used in this study were not highly correlated as the reported correlation values were below 0.8 (Cohen, 1988). Hence, all variables were considered in the study for initial machine learning model development. Table 2 shows the variable importance results generated from the RF variable importance method. Variable importance was ranked based on the percentage of increase in the mean square error (MSE) value. The MSE value presented in Table 2 was averaged over ten repetitions. A variable deemed important had a higher percentage of increase in MSE value (Genuer, Poggi & Tuleau-Malot, 2010). The findings from this study indicate that the variable most associated with adherence (Specific Concern) showed an MSE percentage increase of 12.67%. The least important variable in this study was antihypertensive medication counseling, which reported the lowest percentage of increase in MSE from the RF variable importance method. Variables such as antihypertensive medication intake, the total number of medications taken per day, the presence of other concomitant diseases, gender, age, duration of antihypertensive medication intake, and antihypertensive medication counseling also reported a low percentage of increase of MSE value, and were thus considered less significant in affecting hypertension medication adherence.

**Table 2:** Variable importance generated from RF variable importance method.

Percentage increase of MSE (%)											
Variables	*k* = 1	*k* = 2	*k* = 3	*k* = 4	*k* = 5	*k* = 6	*k* = 7	*k* = 8	*k* = 9	*k* = 10	Average
Specific concern	14.46	10.30	10.75	12.49	15.39	14.88	4.41	11.48	14.00	18.00	12.67
Monthly income	9.81	6.56	9.77	6.41	14.03	6.59	6.11	9.39	7.21	6.39	8.23
General overuse	7.03	12.40	8.39	8.50	5.33	8.79	2.32	10.13	0.09	9.18	7.22
Marital status	7.71	6.95	4.22	15.20	2.94	11.50	9.17	6.70	−0.85	4.10	6.76
Educational level	1.94	3.64	3.71	2.67	4.79	−2.56	4.76	4.67	6.82	11.03	4.15
General harm	1.38	−0.46	7.48	2.04	0.04	8.66	9.67	5.50	−0.16	0.60	3.48
Occupational field	4.21	3.54	4.89	2.23	2.81	1.31	3.09	4.70	−2.39	7.20	3.15
Ethnicity	3.54	4.28	2.89	1.14	6.43	3.85	1.19	2.39	3.64	0.56	2.99
Specific necessity	−1.56	5.91	0.92	−3.04	2.24	−3.71	6.00	0.19	−0.48	1.20	0.77
Religion	0.79	−0.28	−1.20	−0.01	2.41	−1.97	−0.37	2.03	0.91	0.39	0.27
Aids in antihypertensive medications intake	−1.13	−4.02	1.08	−2.49	−3.42	5.21	0.47	2.21	−3.34	−1.37	−0.68
Total number of antihypertensive medications taken per day	−4.91	−3.38	−1.27	−0.05	−1.55	−6.42	−2.54	−0.36	−3.17	−3.56	−2.72
Presence of other concomitant diseases	−3.51	−3.09	−3.78	−2.46	−0.06	−5.68	−5.15	−3.58	−1.43	1.08	−2.77
Gender	−3.99	−4.02	−1.93	−3.10	−2.63	−5.12	−4.13	−2.35	−1.81	−3.70	−3.28
Age	−4.36	−4.24	−3.70	−3.78	−1.12	−2.88	−4.03	−6.18	−5.81	−4.23	−3.93
Duration of antihypertensive medications intake	−6.05	−4.20	−5.89	−5.40	−2.18	−2.72	−3.37	−6.43	−2.74	−1.98	−4.09
Counseling for antihypertensive medications	−5.61	−8.42	−5.70	−4.19	−7.62	−6.35	−8.02	−6.34	−6.20	−0.81	−5.93

**Figure 1:**
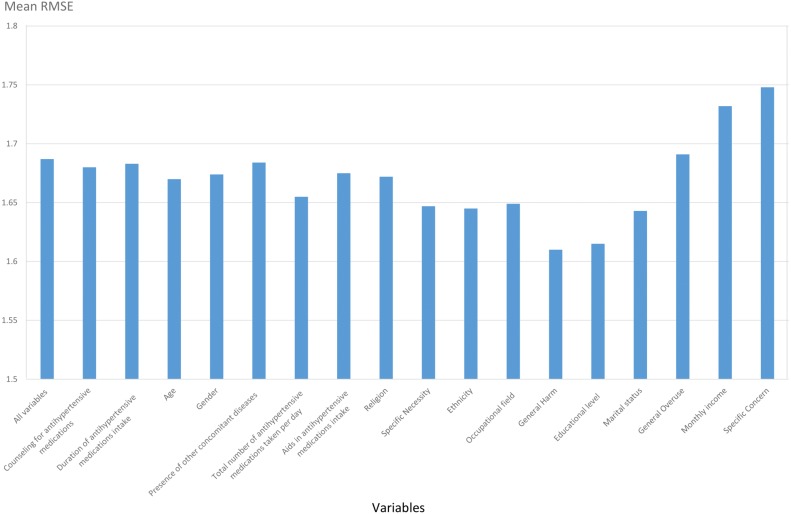
Feature selection process using backward elimination. Each variable indicates increase or decrease in RMSE value upon backward elimination using RF method.

The variables were then ranked in importance according to the increase in their percentage of MSE, and backward elimination was carried out for feature selection. Variables were eliminated in ascending order, and the RMSE of the RF model was determined upon elimination. Figure 1 illustrates five variables that displayed an increase in the RMSE value of the trained RF model upon deletion of the ranked variables: educational level, marital status, General Overuse, monthly income, and Specific Concern. These significant variables identified from the feature selection step were used to develop all the machine learning algorithms.

**Figure 2:**
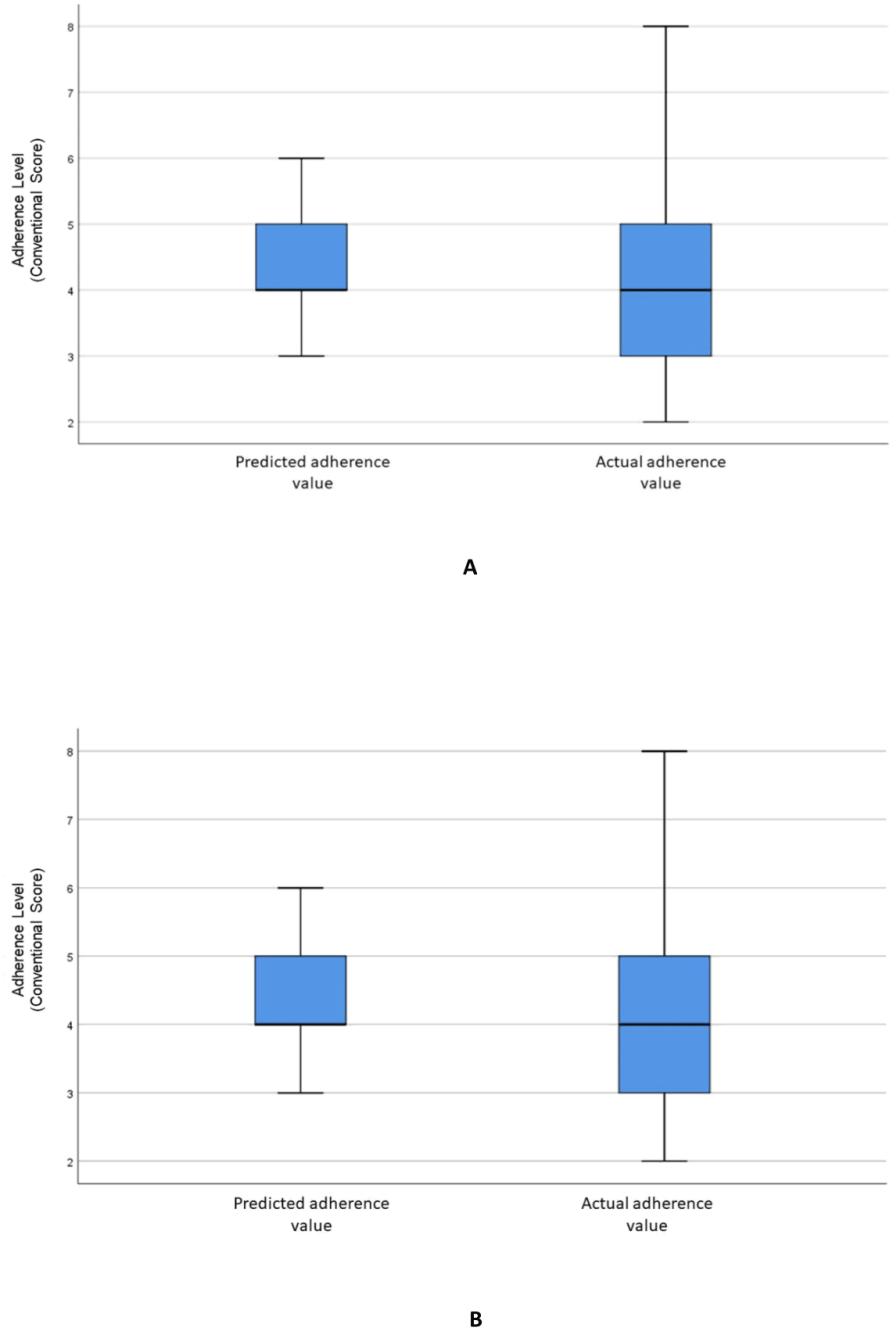
Distribution of the predicted and actual adherence value for RF model. Boxplot of the adherence value distribution for the RF model with (A) all the variables and (B) the selected variables.

**Figure 3:**
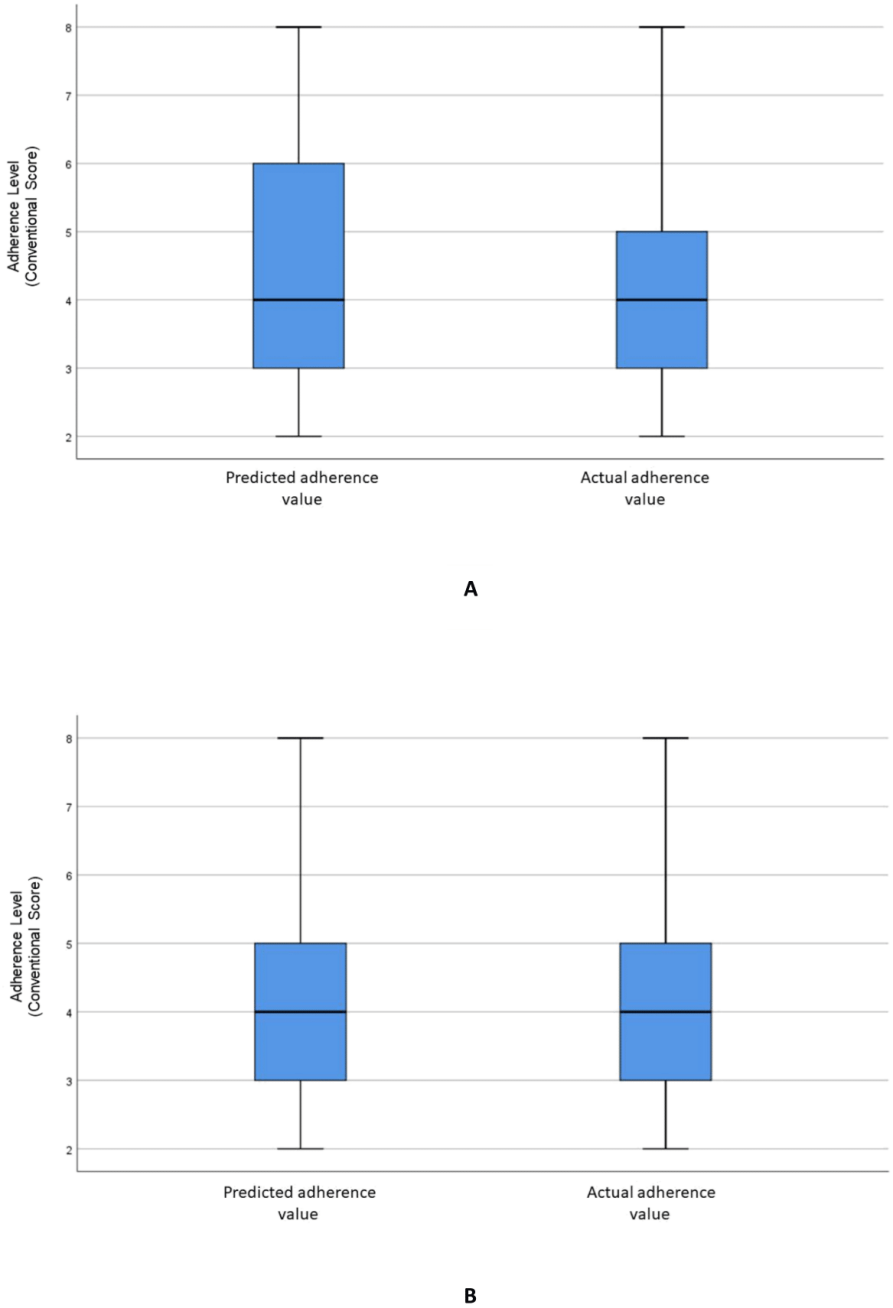
Distribution of the predicted and actual adherence value for ANN model. Boxplot of the adherence value distribution for the ANN model with (A) all the variables and (B) selected variables.

**Figure 4:**
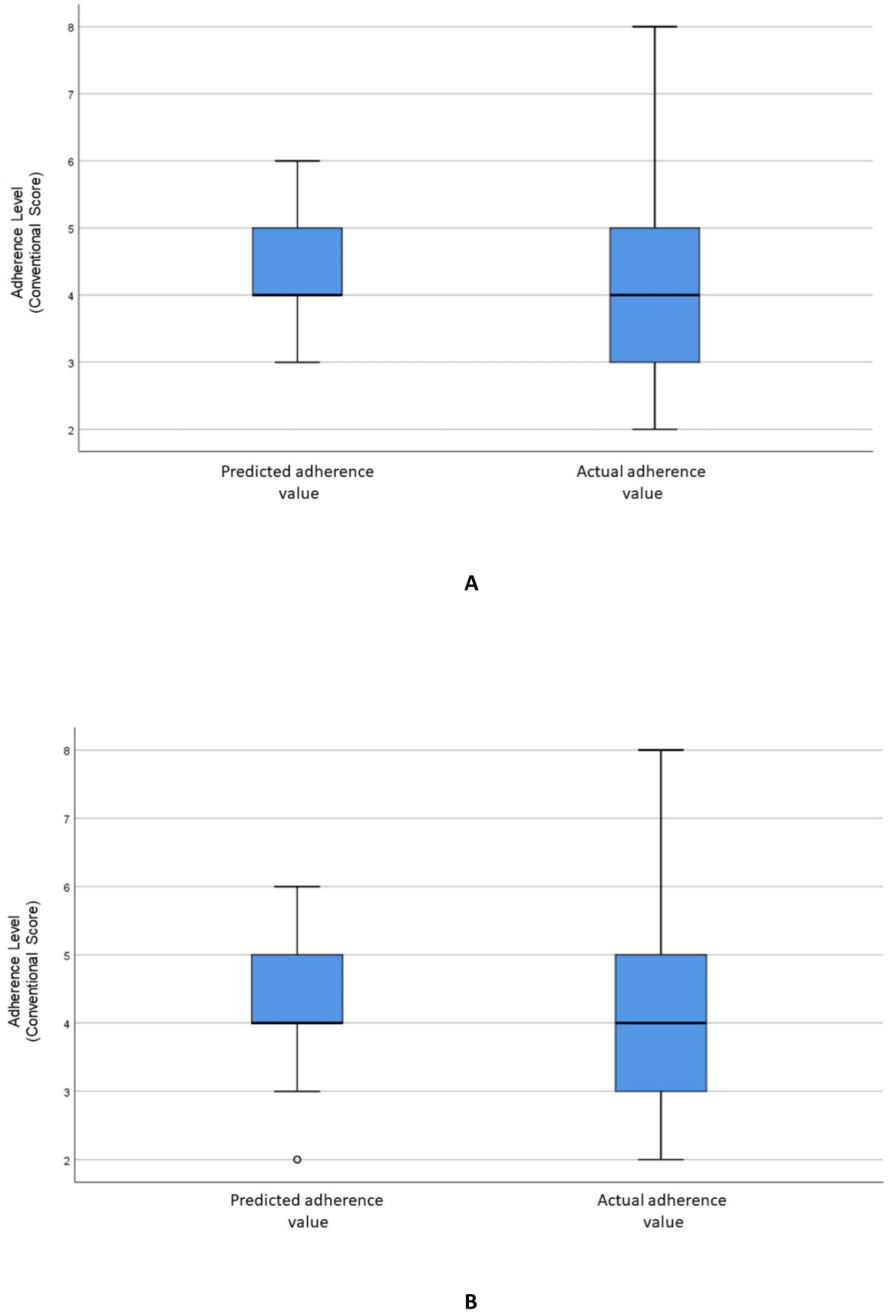
Distribution of the predicted and actual adherence value for SVR model. Boxplot of the adherence value distribution for the SVR model with (A) all the variables and (B) the selected variables.

Figure 2A visualizes the distributions of the predicted and actual values of adherence using the RF model for all variables. Figure 2B illustrates the distributions of the predicted and actual values of adherence for selected variables using the RF model. Figure 3A shows the distributions of actual and predicted values for the ANN model for all variables, and Fig. 3B shows the same for the selected variables. Figure 4A illustrates the actual and predicted value distributions of the SVR model for all variables, and Fig. 4B shows the same for the selected variables. Table 3 shows performance measures for the models developed in this study.

**Figure 5:**
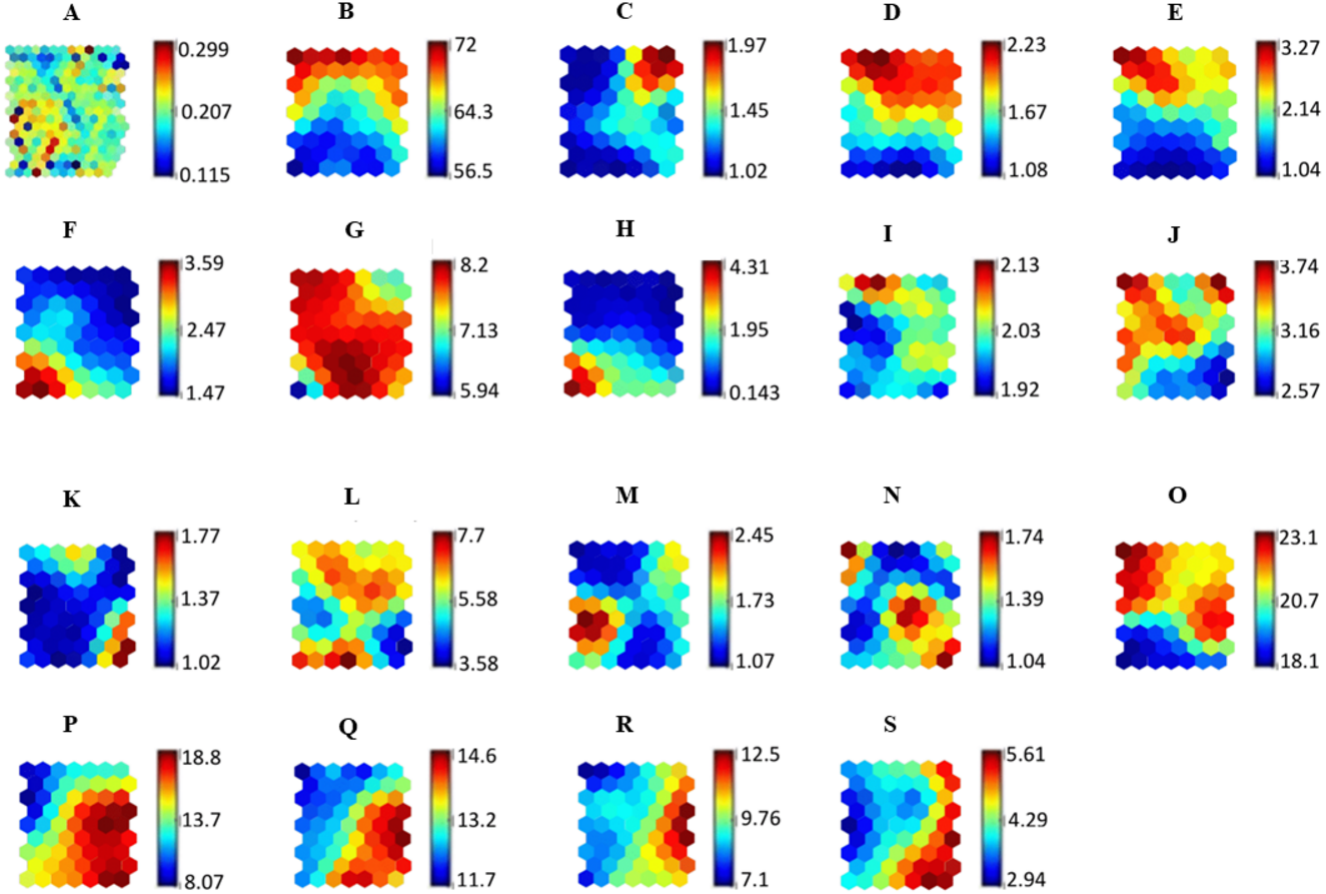
SOM representing the relationship of all variables against the adherence level. The relationship between SOM component plane forms clusters that are represented in the (A) *U*-matrix. Each component plane in the SOM represents variables used in the study which are (B) Age, (C) Gender, (D) Ethnicity, (E) Religion, (F) Educational level, (G) Occupational field, (H) Monthly income, (I) Marital status, (J) Duration of antihypertensive medications intake, (K) Presence of other concomitant diseases, (L) Total number of antihypertensive medications taken per day, (M) Aids in antihypertensive medications intake, (N) Counseling for antihypertensive medications, (O) Specific necessity, (P) Specific concern, (Q) General overuse, (R) General harm and (S) Adherence level.

Figure 5 illustrates the SOM relationship with adherence levels using all input variables associated with the adherence levels of hypertensive patients in regard to medication. The final quantization and topographic errors reported were 0.27 and 0.02, respectively.

## Discussion

In this study, RF permutation importance was used to determine variable importance. RF permutation importance is a more reliable indicator than the Gini impurity function, which is not suitable for predictor variables with many categories (Strobl et al., 2007). The RF permutation importance method, however, will overestimate the importance of variables that are highly correlated with each other (Strobl et al., 2008). In this study, predictor variables were not found to be strongly correlated, justifying the decision to use the RF variable importance method to identify significant variables associated with adherence levels.

**Table 3:** Summary of the result for each of the machine learning model.

Method	Type	RMSE	Accuracy (%)	Sensitivity	Specificity	Wilcoxon (*p*-value)
SVR	All variables	1.71	79.25	0.17	0.96	0.52
	Selected variables	1.55	79.24	0.17	0.93	0.21
RF	All variables	1.62	81.13	0.14	0.95	0.72
	Selected variables	1.53	77.99	0.13	0.98	0.68
ANN	All variables	1.58	53.46	0.33	0.59	0.09
	Selected variables	1.42	64.78	0.15	0.78	0.50

An increase in the RMSE value when a variable is eliminated using a backward elimination procedure indicates that the variable is significant to the adherence levels in hypertensive patients. It was noted that the RMSE increased upon elimination of the following variables in this study: educational level, marital status, General Overuse, monthly income, and Specific Concern.

The RMSE value is used to evaluate machine learning model performance, and it can be minimised by adjusting model parameters. Penalizing significant errors through the defined least-square terms proves to be very useful in improving model performance, especially when calculating model error sensitivities or for data assimilation applications (Chai & Draxler, 2014). There was no significant difference in the RMSE values reported between all methods used in this study. The Wilcoxon signed-rank test for machine learning methods did not show a statistically significant difference between the actual and predicted values for all models (*p* > 0.05), thus providing no evidence for biases in the predicted values. Dimensionality reduction of variables using RF variable importance was noted to improve the performance of the RF, ANN and SVR methods in this study. ANN predictive performance was observed to improve with dimensionality reduction of the dataset (Kauffman & Jurs, 2001). The RF method has the ability to generate the ranked list of variable importance, which is not possible with other methods such as k-NN, SVM, and ANN (Archer & Kimes, 2008). RF is also suitable to be used on small datasets and has been successfully applied in finding essential predictors in the clinical fields (Hsich et al., 2011).

Limited literature is available on the use of RF, SVR, and ANN for regression in studying hypertensive patients’ adherence towards medications. The available articles on hypertensive medication adherence focus on the application of machine learning methods for classification problems. For example, Lee et al. (2013) conducted a study on the adherence of elderly patients with chronic diseases using support vector machines (SVM) for classification, reporting an accuracy of 97.3%. However, the same dataset had been used for testing and training the model, leading to a biased outcome. The study did not use multiple adherence assessment methods for a more comprehensive assessment of adherence level. Son et al. (2010) applied SVM for the prediction of medication adherence in heart failure patients with an accuracy of 77.6%. Our current study used data validation techniques to avoid biases in the obtained results.

However, determining adherence levels should not be limited by dichotomous (adherent versus non-adherent) parameters (Vrijens et al., 2017). Measuring adherence levels using a precise numeric score is vital in differentiating between patients who scored poorly compared to others; hence, a regression-based approach was used in the study.

The methods of assessing medication adherence can be classified by direct and indirect methods of measurement. Measuring drug concentrations or its metabolite in blood or urine are examples of the direct method, which is the most accurate but invasive method. Indirect methods include patient questionnaires, pill counts, rates of prescription fills, assessment of the patient’s clinical outcome, and electric medication monitors. Each method has its strengths and weaknesses, and one of them could be a reference standard for another approach. One of the most frequently used patient questionnaires for the assessment of medication adherence is the Morisky Medication Adherence Scale (MMAS). MALMAS, was developed in Malaysia with minor amendments made to suit the local setting. This method is based on patient recall and may include bias based on patient response. Since medication adherence is a complex multifactorial behavior, it is crucial to ensure an accurate and practical tool for measuring medication adherence is used in routine medical practice. Although clinical decision making is dichotomous or binary, there is a strong reason to use the exact value outcome produced by using a regression type of machine learning method. Based on the findings in a systematic review and meta-analysis of the Morisky Medication Adherence Scale-8 (MMAS-8) by Moon et al. (2017) looking at the accuracy of a screening tool for medication adherence, nearly half of all studies dealing with diagnostic accuracy had a reference standard with high risk or unclear descriptions in both the assessment of risk of bias and applicability. It has been reported that Cronbach’s alpha is not appropriate for the internal consistency and reliability of a test, but it was used by all studies included in this review (Moon et al., 2017). Despite that, Moon et al. (2017) highlighted that individual studies calculating sensitivity and specificity used the cut-off value of 6 as suggested by Morisky, although MMAS-8 is under development. If they had addressed sensitivity and specificity using different cut-off values, more information about the diagnostic accuracy of MMAS-8 could have been provided. During its development, Morisky suggested 6 as a cut-off value in MMAS-8, so most studies show criterion validity outcomes using the cut-off value of 6. Since there are weaknesses noted with the accuracy of the assessment method used above, alternative dichotomous methods such as machine learning with a larger sample size should be used in the future to compare results. We expect that comparing the conventional adherence instrument with the suggested alternative method will elucidate any inconsistencies of either method.

Machine learning models are considered to be black-box models. In this study, we have shown that clinical data can be visualized in a 2-dimensional representation using the SOM technique. A SOM was used to visualize higher dimensional nonlinear data and feature distribution in the unified distance matrix map to better understand the relationship between variables associated with adherence levels in hypertensive patients. Medication adherence is a complex phenomenon with many correlating causes. This study identified several factors associated with patients’ adherence to antihypertensive medicines. These factors (Specific Concern, General Overuse, marital status, educational level, and monthly income) are visualized using a SOM to explain their underlying relationships.

The SOM component plane illustrates that patients with a high Specific Concern score (more than 15) are associated with a lower adherence level (score of 4.0) towards hypertensive medication. Jamous et al. (2014) reported that Specific Concern is associated with patients’ adherence to the intake of antihypertensive medication due to the limited amount of information received by the patients regarding potential problems and side effects of their medications. Patients identified with high Specific Concern felt that every medication prescribed by the doctors had a negative side effect, resulting in their non-adherence to the prescribed medication.

General Overuse refers to the belief that doctors overprescribe medication, leading to medication overdoses. This belief causes patients to be less adherent to antihypertensive medications. Higher General Overuse scores (more than 12) are related to a lower adherence value (less than 6.0), as illustrated in the SOM component plane map. This finding is similar to those of Horne & Weinman (1999), and Jamous et al. (2014).

The SOM component plane illustrates how patients with high Specific Necessity and high General Overuse are associated with low adherence. This result is similar to that of a study by AlHewiti (2014), where patients with high General Overuse and Specific Concerns had low adherence levels.

Understanding the relationships between adherence and demographic factors is essential because of the diversity and complexity of medication intake behavior. In our study, as illustrated in the SOM, demographic factors such as marital status, educational level and monthly income are associated with antihypertensive medication adherence levels. The present study shows that unmarried people have low adherence levels of 2.0–4.0. Jin et al. (2008) and Wu et al. (2012) found that marital status positively affects a patient’s adherence towards medication intake.

The SOM component plane demonstrates that patients with a monthly income of less than RM1000 are more likely to be adherent to medication. This finding is similar to a study by Kassahun et al. (2016).

The SOM component plane also shows that patients with low educational level were more adherent to their medication. This could be due to the social desirability bias in which patients with low literacy perceive medication information differently leading to higher adherence levels, as highlighted by AlHewiti (2014), Alkatheri & Albekairy (2013) and Kassahun et al. (2016).

We expect that our findings from this study will provide effective alternatives to conventional methods in understanding hypertensive patients’ adherence levels, and will allow the creation of valuable adherence educational programs.

Machine learning methods are suitable for identifying complex interactions between patient characteristics and adherence. Furthermore, machine learning methods have other appealing features; for instance, the selection of important variables does not require specific criteria (Lunetta et al., 2004).

Complex interactions or nonlinear relationships can also be explained using statistical methods. Nonlinear effects can be modelled using splines and fractional polynomials in addition to simple polynomial terms. Similar to statistical methods, machine learning algorithms can automatically select variables. However, statisticians usually avoid stepwise methods as these have adverse effects in regard to biases and overstated statistical importance. It is important for clinicians to understand or have an insight into clinical model development. Machine learning models are considered ‘black boxes,’ and this can be considered a limitation of the study. Black box approaches are not suitable for clinical decisions that generally require the model to be validated in a manner that is relatively simple for regression models, but not for most machine learning methods. The application of SOM in this study has shown that clinical data can be visualized in a 2-dimensional representation. SOM allows for the discovery of relationships among variables that are associated with adherence.

Future enhancements to this study should include a comparison between ML methods and conventional modelling approaches, such as logistic regression, in order to confirm machine learning applicability in determining hypertension medication adherence.

The complementary method found in this study can be used to confirm the specificity and sensitivity of different methods used to predict factors affecting medication adherence among hypertensive patients. These findings will enable healthcare practitioners to identify high-risk patients who are more likely to be non-adherent towards their medications. Using this method, we can obtain exact adherence scores that are useful in targeting a specific group of patients. Specific intervention can then ensure that this high-risk group of patients is given extra attention to ensure higher adherence towards their medications.

## Conclusion

This type of study has not yet been reported in the literature and can be considered novel as it uses a combination of machine learning methods in determining medication adherence in hypertensive patients. We have identified five important variables associated with hypertensive patients’ adherence levels: Specific Concern, General Overuse, marital status, monthly income and educational level. Applying a combination of RF and SOM techniques showed the suitability of the potential tool for the selection of significant variables, prediction, and visualization. It is evident from this work that it is possible to create a compressed data representation when the abundance of data obscures straightforward diagnostic reasoning. Identifying patients with low adherence levels can be used to create educational or counseling strategies advising the importance of hypertension medication in managing the disease. At this stage, it is not yet possible to claim the results have universal application since it is based upon limited clinical data. If it is used within a validation system and continually recreated as more data are collected, clinicians can assess the particular risks to their patients.

##  Supplemental Information

10.7717/peerj.8286/supp-1 Code S1 Machine learning RF code for all the variables

10.7717/peerj.8286/supp-2 Code S2 Machine learning RF code for selected variables

10.7717/peerj.8286/supp-3 Code S3 Machine learning ANN code for all the variables

10.7717/peerj.8286/supp-4 Code S4 ANN code for selected variables Machine learning ANN code for selected variables

10.7717/peerj.8286/supp-5 Code S5 Machine learning SVR code for all the variables

10.7717/peerj.8286/supp-6 Code S6 Machine learning SVR code for selected variables

10.7717/peerj.8286/supp-7 Dataset S1 Raw data for hypertension patient adherence level

10.7717/peerj.8286/supp-8 Supplemental Information 1 RF dataset for all variables

10.7717/peerj.8286/supp-9 Supplemental Information 2 RF dataset for selected variables

10.7717/peerj.8286/supp-10 Supplemental Information 3 The raw data for ANN with all the variables

10.7717/peerj.8286/supp-11 Supplemental Information 4 The raw data for ANN with selected variables

10.7717/peerj.8286/supp-12 Supplemental Information 4 The raw data for SVR with all the variables

10.7717/peerj.8286/supp-13 Supplemental Information 5 The raw data for SVR with selected variables

10.7717/peerj.8286/supp-14 Questionnaire S1 Questionnaire

## Additional Information and Declarations

### Data Availability

The following information was supplied regarding data availability: The raw data and code are available in the Supplemental Files.
