# Supplementary material for: Determining hypertensive patients’ beliefs towards medication and associations with medication adherence using machine learning methods
Source: PeerJ. 2020 Mar 13;8:e8286. doi: 10.7717/peerj.8286 (PMC7075362; doi:10.7717/peerj.8286)
Supplement: Questionnaire S1 [file peerj-08-8286-s014.docx]

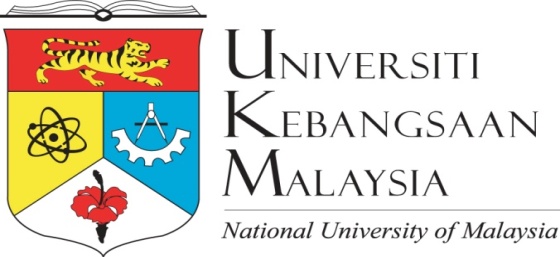


**Faculty of Pharmacy**

**KAJIAN SOAL SELIDIK**

***SURVEY***

**Kajian Kepercayaan Pesakit Dalam Mempengaruhi Kepatuhan Mereka Terhadap Pengambilan Ubat-Ubatan Di Kalangan Pesakit Hipertensi**

***A Study Exploring Patients’ Belief In Influencing Their Medication Adherence Among Hypertensive Patients***

**Arahan Umum:**

1. **Soal selidik ini mengambil masa lebih kurang 20 minit. Sila luangkan masa untuk menjawab soalan-soalan yang disediakan. Pendapat yang jujur adalah penting untuk menjayakan penyelidikan ini.**
2. **Penyertaan anda dalam kajian ini adalah amat dihargai. Sekiranya anda mempunyai sebarang pertanyaan tentang kajian ini, sila hubungi saya.**

**Nama : WONG MEE SIENG**

**No. Telefon : 0198592814**

**Email : wms88heart@hotmail.com**

**Jawapan anda akan dikendalikan secara sulit dan maklumat peribadi tidak akan dimasukkan dalam laporan kajian atau penerbitan.**

***GENERAL DIRECTION:***

1. ***This survey will take about 20 minutes. Please spare few minutes to answer the following questions. Your honest opinion is important to ensure the success of this study.***
2. ***Your participation in this study would be highly appreciated. If you have any questions regarding the survey, please do not hesitate to contact me.***

***Name: WONG MEE SIENG***

***Phone No. : 0198592814***

***Email : wms88heart@hotmail.com***

***Your responses will be handled confidentially and information that may identify you will not be included in the study reports or publications.***

| **Bahagian A: Demografik**  ***Section A: Demographic***  Sila tandakan (√) pada petak yang disediakan dan isikan jawapan pada ruang yang berkenaan.  *Please tick (√) in the box below and fill in the answer in the appropriate space.* | | |
| --- | --- | --- |
| RN / Nama:  *RN/ Name:* | Umur:  *Age :* | |
| Jantina:  *Gender:*  Lelaki Perempuan  *Male Female* | Kaum: Melayu Cina  *Ethnicity: Malay Chinese*  India Lain-lain  *Indian Others* | |
| Agama: Islam Buddha  *Religion: Islam Buddha*  Hindu Kristian  *Hindu Christian*  Lain-lain  *Others* | Tahap pengajian:  *Educational level:*  Rendah Menengah  *Primary Secondary*  Tinggi Sarjana  Muda  *Tertiary Degree*    Pasca siswazah Doktor  *Master* Falsafah  *Doctor of*  *PhilosophyMaster* | |
| Bidang Pekerjaan:  *Occupation field:*  Pertanian Perniagaan  *Agriculture Business*  Pendidikan Kesihatan  *Education Health*  Kerjarumah Kejuruteraan  *Housework Engineering* | Tidak Bekerja Pesara  *Unemployed Retiree*  Lain-lain  Sila nyatakan: _________________  *Others*  *Please specify:_________________* | |
| Pendapatan sebulan:  *Monthly income:*  < RM 1,000  RM 1,000-RM 2,000  RM 2,000-RM 3,000  RM3,000-RM 4,000  RM 4,000-RM 5,000  > RM 5,000 |  | Status perkahwinan:  *Marital status:*    Bujang Sudah berkahwin  *Single Married*  Janda Duda  *Widow* *Widower* |

| Berapa lamakah bagi pengambilan ubat hipertensi ?  *Taking hypertensive medications since when?*  Kurang daripada 1 tahun 5-10 tahun  *Less than 1 year* *5-10 years*  1-5 tahun Lebih daripada 10 tahun  *1-5 years* *More than 10 years* | |
| --- | --- |
| Adakah menghidapi penyakit yang lain selain daripada penyakit hipertensi ?  *Suffering for any diseases apart from hypertension ?*    Ya Kalau ya, sila nyatakan penyakit apa. Tidak  *Yes If yes, please state the disease. No*  *__________________________________* | |
| Ubat hipertensi yang diambil:  *Hypertensive medication (s) taken:* | Ubat-ubatan lain yang digunakan:  *Concomitant medicines:* |
| Bilangan ubat yang diambil sehari (termasuk ubat hipertensi dan juga ubat-ubatan yang lain):  *Total number of medication (s) taken per day( included hypertensive medications and concomitant medicines:* | |
| Bantuan dalam pengambilan ubat hipertensi :  *Aids in hypertensive medication(s) taking:*  Bekas pil Jadual waktu Lain-lain (sila nyatakan)………..  *Pill box Timetable Others (Please specify)…………..* | |
| Pernah diajar tentang pengambilan ubatan hipertensi :  *Received counseling for hypertensive medication(s) taking:*    Ada Kalau ada, sila nyatakan siapa? Tidak  *Yes*  *If yes, who? No*    __________________________ | |

| **Bahagian B : Pandangan anda tentang ubat yang dipreskripsikan kepada anda.**  ***Section B : Your views about medicines prescribed for you.***  Kami ingin mendapatkan pandangan peribadi anda terhadap ubat yang dipreskripsi kepada anda. Dengan menggunakan skala 1 ‘sangat tidak setuju’ hingga 5 ‘sangat setuju’ sila berikan pandangan anda dengan menandakan(√) pada petak berkenaan.  *We would like to ask your personal opinions about medicines that are being prescribed to you. By using the scale of 1 ‘strongly disagree’ to 5 ‘strongly agree’ please indicate your opinion by ticking (√) in the appropriate box.* | | | | | | |
| --- | --- | --- | --- | --- | --- | --- |
| **Skala:** 1 = ‘Sangat tidak setuju’ 2 = ‘Tidak setuju’ 3 = ‘Tidak pasti’  *Scale： Strongly disagree Disagree Not sure*    4 =‘Setuju’ 5 = ‘Sangat setuju’ *Agree Strongly agree* | | | | | | |
| **Fakta-fakta tentang ubat-ubatan yang dipreskripsi secara umum untuk pesakit.**  ***Statements about general medications prescribed for the patient.*** | | | | | | |
|  |  | 1 | 2 | 3 | 4 | 5 |
| 1 | Tanpa ubat-ubatan, doktor kurang mampu menyembuhkan individu .  *Without medicines doctors would be less able to cure people.* |  |  |  |  |  |
| 2 | Ubat-ubatan baru lebih berkesan berbanding dengan ubat-ubatan lama.  *Newer medicines are more effective than older ones.* |  |  |  |  |  |
| 3 | Kebanyakan ubat-ubatan akan menyebabkan ketagihan.  *Most medicines are addictive.* |  |  |  |  |  |
| 4 | Individu yang mengambil ubat-ubatan perlu menghentikan rawatan mereka untuk sementara.  *People who take medicines should stop their treatment for a while.* |  |  |  |  |  |
| 5 | Ubat-ubatan hanya berkesan jika mereka mengambil ubatan secara tetap.  *Medicines only work if they are taken regularly.* |  |  |  |  |  |
| 6 | Ubat-ubatan mendatangkan lebih kemudaratan daripada kebaikan.  *Medicines do more harm than good.* |  |  |  |  |  |
| 7 | Ubat-ubatan bukan remedi semulajadi.  *Medicines are not natural remedies.* |  |  |  |  |  |
| 8 | Semua ubat-ubatan adalah racun.  *All medicines are poisons.* |  |  |  |  |  |
| 9 | Ketiadaan ubat-ubatan adalah lebih baik.  *It is better to do without medicines.* |  |  |  |  |  |
| 10 | Bahan-bahan semula jadi adalah lebih selamat berbanding dengan ubat-ubatan.  *Natural remedies are safer than medicines.* |  |  |  |  |  |
| 11 | Ubat-ubatan yang lebih kuat adalah lebih berbahaya berbanding dengan ubat-ubatan yang lebih lemah.  *Stronger medicines are more dangerous than weaker medicines.* |  |  |  |  |  |
| 12 | Ubat-ubatan adalah amat memudaratkan.  *Medicines are a necessary evil.* |  |  |  |  |  |
| 13 | Doktor terlalu percaya terhadap ubat-ubatan.  *Doctors place too much trust on medicines.* |  |  |  |  |  |
| 14 | Jika doktor dapat meluangkan masa yang lebih dengan pesakit, mereka akan kurang mempreskripsi ubat-ubatan.  *If doctors had more time with patients they would prescribe fewer medicines.* |  |  |  |  |  |
| 15 | Terdapat perbezaan yang ketara di antara ubat dengan dadah.  *There is a big difference between a medicine and drug.* |  |  |  |  |  |
| 16 | Ubatan-ubatan yang diperoleh adalah lebih penting berbanding dengan doktor yang dijumpai.  *The medicine you get is more important than the doctor you see.* |  |  |  |  |  |
| 17 | Doktor menggunakan terlalu banyak ubat-ubatan.  *Doctors use too many medicines.* |  |  |  |  |  |
| 18 | Kebanyakan ubat-ubatan adalah selamat.  *Most medicines are safe.* |  |  |  |  |  |

| **Fakta-fakta tentang ubat-ubatan yang dipreskripsikan secara spesifik untuk pesakit hipertensi.**  ***Statements about specific hypertensive medications prescribed for the patient.*** | | | | | | |
| --- | --- | --- | --- | --- | --- | --- |
|  |  | 1 | 2 | 3 | 4 | 5 |
| 19 | Ubat saya mengawal penyakit hipertensi saya.  *My medication controls my hypertension.* |  |  |  |  |  |
| 20 | Tanpa ubat, saya akan berasa sangat sakit.  *Without my medicines, I would be very ill.* |  |  |  |  |  |
| 21 | Kesihatan saya sekarang bergantung kepada ubat hipertensi saya.  *My health, at present, depends on hypertensive medication.* |  |  |  |  |  |
| 22 | Ubat mencegah penyakit hipertensi saya daripada menjadi lebih teruk.  *My medication prevents my hypertension from becoming worst.* |  |  |  |  |  |
| 23 | Kesihatan saya pada masa depan akan bergantung kepada ubat hipertensi saya.  *My health in the future will depend on my hypertensive medication.* |  |  |  |  |  |
| 24 | Kadang-kala saya berasa bimbang akan kesan jangka panjang ubat hipertensi saya.  *I sometimes worry about the long term effects of hypertensive medication.* |  |  |  |  |  |
| 25 | Pengambilan ubat hipertensi membimbangkan saya.  *Having to take hypertensive medication worries me.* |  |  |  |  |  |
| 26 | Kadang-kala, saya berasa bimbang akan terlalu bergantung kepada ubat-ubatan hipertensi saya.  *I sometimes worry about becoming too dependent on hypertensive medication.* |  |  |  |  |  |
| 27 | Ubat-ubatan hipertensi menganggu kehidupan saya.  *Hypertensive medication disrupts my life.* |  |  |  |  |  |
| 28 | Ubat-ubatan hipertensi saya adalah satu misteri bagi saya.  *Hypertensive medications are a mystery to me.* |  |  |  |  |  |
| 29 | Kehidupan saya tidak dapat diteruskan sekiranya tiada ubat-ubatan hipertensi saya.  *My life would be impossible without my hypertensive medicines.* |  |  |  |  |  |
| 30 | Ubat-ubatan hipertensi saya sangat berkesan.  *My hypertensive medicines are powerful.* |  |  |  |  |  |
| 31 | Saya ingin menukar ubat-ubatan saya sekarang.  *I would like to change my present treatment.* |  |  |  |  |  |
| 32 | Adalah sukar bagi saya untuk mengambil ubat hipertensi saya seperti apa yang telah diarahkan oleh doktor.  *It is difficult for me to take my hypertensive medicines in exactly the way my doctor told me.* |  |  |  |  |  |
| 33 | Saya boleh mengawal keadaan hipertensi saya tanpa ubat-ubatan.  *I can cope with my hypertensive condition without medicines.* |  |  |  |  |  |
| 34 | Keadaan penyakit hipertensi saya terkawal dengan ubat-ubatan.  *I am in control for hypertension with my medications.* |  |  |  |  |  |

| **Bahagian C: Kepatuhan terhadap ubat-ubatan**  ***Part C : Adherence Scale*** | | | | |
| --- | --- | --- | --- | --- |
| Sila jawab setiap soalan berdasarkan pengalaman anda dalam pengambilan ubat-ubatan hipertensi anda.  *Please answer each question based on your personal experience with your hypertensive medications.* | | | | |
| **Sila tandakan** ( **√ ) SATU jawapan untuk setiap soalan yang disediakan.**  ***Please tick ( √ ) ONE answer for each of the questions below.*** | | | | |
|  |  | | **Tidak/ *No***  **= 0** | **Ya/ *Yes***  **= 1** |
| 1 | Adakah anda kadangkala terlupa untuk mengambil ubat hipertensi anda ?  *Do you sometimes forget to take your hypertensive pills?* | |  |  |
| 2 | Kadangkala pesakit tidak mengambil ubat mereka dengan alasan lain selain terlupa. Cuba fikirkan sepanjang dua minggu yang lepas, adakah anda tidak mengambil ubat hipertensi anda?  *People sometimes miss taking their medications for reasons other than forgetting. Thinking over the past two weeks, were there any days when you did not take your hypertensive medicine?* | |  |  |
| 3 | Pernahkah anda mengurangkan atau berhenti untuk mengambil ubat anda tanpa memberitahu doktor kerana anda berasa tidak selesa apabila mengambil ubat tersebut ?  *Have you ever cut back or stopped taking your medication without telling your doctor, because you felt worse when you took it?* | |  |  |
| 4 | Apabila anda dalam perjalanan atau meninggalkan rumah, adakah anda kandangkala terlupa untuk membawa ubat hipertensi anda?  *When you travel or leave home, do you sometimes forget to bring along your hypertensive medication?* | |  |  |
| 5 | Adakah anda mengambil ubat hipertensi anda semalam?  *Did you take your hypertensive medicine yesterday?* | |  |  |
| 6 | Bila anda merasa bahawa keadaan hipertensi anda berada di bawah kawalan, adakah anda kadangkala berhenti mengambil ubat anda?  *When you feel like your hypertensive condition is under control, do you sometimes stop taking your medicine?* | |  |  |
| 7 | Mengambil ubat setiap hari menimbulkan ketidakselesaan bagi sesetengah individu. Adakah anda pernah merasa terganggu mengikuti pelan rawatan hypertensi anda?  *Taking medication everyday is a real inconvenience for some people. Do you ever feel hassled about sticking to your hypertensive treatment plan?* | |  |  |
| 8 | Berapa kerapkah anda mengalami kesulitan untuk mengingati pengambilan kesemua ubat anda? **(Sila tandakan (√) jawapan yang paling sesuai)**  *How often do you have difficulty remembering to take all your medications?* ***(Please tick (*√) the most appropriate answer *)*** | | | |
|  | Tidak Pernah / Jarang  *Never/Rarely* |  | | |
|  | Pernah sesekali  *Once in a while* |  | | |
|  | Kadang-kala  *Sometimes* |  | | |
|  | Kerap  *Usually* |  | | |
|  | Hampir setiap waktu  *All the time* |  | | |

******************* Soalan tamat *******************

*End of question*

Terima kasih kerana menjawab soalan soal selidik ini

*Thank you for your cooperation to fill the questionnaire*
